# Prophage Activation in the Intestine: Insights Into Functions and Possible Applications

**DOI:** 10.3389/fmicb.2021.785634

**Published:** 2021-12-13

**Authors:** Jie Hu, Hao Ye, Shilan Wang, Junjun Wang, Dandan Han

**Affiliations:** State Key Laboratory of Animal Nutrition, College of Animal Science and Technology, China Agricultural University, Beijing, China

**Keywords:** gut microbes, prophage activation, intestinal health, microbial regulation, phage therapy

## Abstract

Prophage activation in intestinal environments has been frequently reported to affect host adaptability, pathogen virulence, gut bacterial community composition, and intestinal health. Prophage activation is mostly caused by various stimulators, such as diet, antibiotics, some bacterial metabolites, gastrointestinal transit, inflammatory environment, oxidative stress, and quorum sensing. Moreover, with advancements in biotechnology and the deepening cognition of prophages, prophage activation regulation therapy is currently applied to the treatment of some bacterial intestinal diseases such as Shiga toxin-producing *Escherichia coli* infection. This review aims to make headway on prophage induction in the intestine, in order to make a better understanding of dynamic changes of prophages, effects of prophage activation on physiological characteristics of bacteria and intestinal health, and subsequently provide guidance on prophage activation regulation therapy.

## Introduction

Bacteriophages represent the majority of intestinal microorganisms, which have been intimately associated with gut health, since they play crucial roles in maintaining intestinal homeostasis, bacterial concentrations, and microbiota diversity, etc. (Hatfull and Hendrix, 2011; Vitetta et al., 2018; Gogokhia et al., 2019). Temperate phages are defined by their life characteristics to switch between the lysogenic and lytic life states, and ultimately affect fitness benefits of the hosts and the function of the entire gut ecosystem (Jover et al., 2013; Obeng et al., 2016; Cornuault et al., 2018). Prophages, viral DNA that originates from temperate phages, have been identified in the genome of approximately 40–50% of microbe (Howard-Varona et al., 2017). A large amount of commensal bacteria in the intestinal tract of C57BL/6J mice are lysogens, of which there are more prophages in Firmicutes and Proteobacteria than in Bacteroidetes and Actinobacteria (Kim and Bae, 2018). Most prophage sequences are integrated into bacterial chromosome, accounting for as much as 20% of host genome (Khan and Wahl, 2019). Particularly, *Escherichia coli* phage P1 and lambda-related phage N15 exists extrachromosomally as a plasmid in a circular form and with hairpin telomeres in a linear form, respectively (Łobocka et al., 2004; Ravin, 2015). Prophages and their hosts coexist and coevolve in intestinal environments (Cornuault et al., 2020).

While most prophages are highly stable, prophages can be specifically activated, leading to the excision of DNA and release of active phages (Oh et al., 2019). There are evidences suggesting that a large number of virus-like particles contained in human gut are derived from prophages instead of lytic phages (Breitbart et al., 2003; Reyes et al., 2012; Howe et al., 2016). Various factors (for example, diet and some commonly used drinks) may activate prophages in the intestine (Pierzynowska et al., 2018; Boling et al., 2020). Both bacteria characteristic and intestinal health have been clarified to be influenced by the activity of prophages, which is of particularly important to understand prophage activation that occurs frequently in the gut (Balasubramanian et al., 2016; Vitetta et al., 2018).

Phage therapy has been used as a medical alternative in certain countries considering its advantage on host specificity, micro-ecological balance, cost, biofilm penetration, wide distribution, and low inherent toxicity over antibiotic treatment. With the in-depth understanding of phages and the development of biotechnology, phage types used in phage therapy are not limited to the lytic phages, but also expanded into the prophages (i.e., prophage activation) (Sheng et al., 2016; Alexander et al., 2019). In the present review, we discuss the current knowledge on the impact factors and mechanisms of prophage activation in the intestine, the spreading of induced active phages in the intestine, effects of prophage activation on physiological characteristics of bacteria and intestinal health, and phage therapy by regulating prophage activation.

## Impact Factors and Mechanisms of Prophage Activation in Gut

### Factors Mediated Prophage Activation in Intestinal Environments

Many studies indicated that diet could alter the composition of gut microbial community (Turnbaugh et al., 2009; Muegge et al., 2011; Boling et al., 2020). The response of gut microbiome to short-term macronutrient intake is rapid, diet-specific, and reproducible. However, this effect can only last in a short period, and as a consequence, bacterial composition will revert to the initial state after the dietary interference (Martínez et al., 2010; David et al., 2014; Howe et al., 2016). The effect of dietary intervention on bacteriophages was found to be more lasting and significant than it on the bacterial communities (Minot et al., 2011). Specifically, the dynamic changes in phage community compositions induced by diet retained long-term consequences (Howe et al., 2016). The prophage metagenome in the murine intestine had high sequence similarity with the free phage genome at the end of consecutive dietary shifts, indicating that most lysogens are active (Kim and Bae, 2018). As described by Boling et al. (2020), clove, artificial sweeteners, grapefruit seed extract, and propolis glycerite etc. were able to manipulate the gut microbiome by activating prophages. Dietary sugar (fructose, galactose, and xylose) promoted phage production in *Lactobacillus reuteri* (*L. reuteri*) (Oh et al., 2019). Although numerous evidences for diet modulation on intestinal phages have been reported, the biological meaning is rarely known. Only one study found that some commonly used drinks enhanced Stx prophage activation in enterohemorrhagic *Escherichia coli* (EHEC), potentially enhancing pathogenicity of the pathogen (Pierzynowska et al., 2018). Further studies are needed to clarify diet-mediated effects.

Most antibiotics cause disruption of prophage maintenance. Prophage activation was observed in *Staphylococcus aureus* treated with β-lactam antibiotics (Maiques et al., 2006). Quinolone antibiotics that caused DNA double-strand breaks were typical prophage inducers (Zhang et al., 2000). Antibiotic-mediated prophage induction is usually intimately associated with dissemination of virulence factors (for example, Shiga toxin) and antibiotic resistant genes in bacteria. Some metabolites of bacteria are also confirmed to be efficient inducers of prophages. Bile acid has been reported to induce prophages in *Salmonella* (Hernández et al., 2012). Short-chain fatty acids (SCFAs) produced by bacterial metabolism or administrated exogenously resulted in increased active phage production in *L. reuteri* 6475, *L. reuteri* ATCC 55730, and *Lactococcus lactis* NZ9000 (Oh et al., 2019). These facts indicate that metabolic status of intestinal microorganisms contributes to reconstruction of microbial community structure (bacteria and viruses).

Furthermore, intestinal environment (i.e., temperature, gastrointestinal transit, intestinal disease, and oxidative stress) has been recognized as a crucial factor in regulating prophage induction. Previous studies indicated that prophage induction could mainly be associated with high temperature (Horiuchi and Inokuchi, 1967). Notably, the dynamic changes in prophages were also observed during bacterial cold stress (Zeng et al., 2016). Oh et al. (2019) demonstrated that the ecological conditions in gut environment affected the phageome. Prophages LRΦ1 and LRΦ2 were identified in strain *L. reuteri* 6475 (Oh et al., 2019). The survival rate of wild-type *L. reuteri* was at least 3.7-fold lower than the level of mutant bacteria without prophages in the cecum, colon, and feces of mice after oral administration of *L. reuteri*. Phage LRΦ1 was predominantly produced in the gut. Thus, prophages could be activated during gastrointestinal transit. Similar results were observed in *L. reuteri* VPL1014 (Alexander et al., 2019). Inflammatory bowel disease (IBD) led to elevated phage number in the intestine (Duerkop et al., 2018). Prophage activation of four *Myoviridae* phages were identified in patients suffering from *Clostridium difficile* infection (CDI) (Meessen-Pinard et al., 2012). Intestinal oxidative stress (for example, hydrogen peroxide) provoked induction of Shiga toxin-carrying lambdoid prophages (Licznerska et al., 2015). Collectively, under the threat of changes in intestinal environments, prophages are generally activated, which possibly alter the gut microbial communities and affect survival of the hosts.

What’s more, free Pf1-like phages were highly abundant when *Pseudomonas aeruginosa* was infected with N4-like lytic podovirus Ab09, suggesting that Pf1-like prophages were largely excised (Latino et al., 2019). Prophage activation occurred in intestinal pioneer bacteria in early infants (1 month old) and led to formation of active phages that occupied a dominant part of viral-like particles (Liang et al., 2020). So far, several pieces of evidences suggest that quorum sensing caused the modification of prophages induction rate, and ultimately affected ecological networks in gut bacterial communities and functional gene distribution (for example, virulence genes) (Rossmann et al., 2015; Liang et al., 2019; Tan et al., 2020). It is also well known that prophage spontaneous activation is universal among lysogenic bacteria, though at low frequency (Bertani, 1951). Additionally, intestinal cells may contribute to the change of phageome structure. For example, cells (such as macrophages)-producing nitric oxide, an antimicrobial defense molecule, was shown to increase Shiga toxin-converting phage production in EHEC (Ichimura et al., 2017).

Altogether, these findings indicate instability of intestinal prophages (Table 1) and a great potential impact of prophage activation on altering the gut microflora. Indeed, almost all tested gut bacteria have been experimentally confirmed to contain active prophages (reviewed in Sausset et al., 2020).

**TABLE 1:** Factors influencing prophage induction in the gut.

Factors	Prophage	Host	References
High and low fat diets	Gut prophages	Gut bacterial communities	Howe et al., 2016
Stevia, clove, and propolis	Unspecified	*Bacteroides thetaiotaomicron* VPI-5482	Boling et al., 2020
Uva ursi, propolis, and aspartame	Unspecified	*Enterococcus faecalis*	Boling et al., 2020
Grapefruit seed extract, stevia, and toothpaste	Unspecified	*Staphylococcus aureus* CA15	Boling et al., 2020
Fructose, galactose, and xylose	LRΦ1 and LRΦ2	*Lactobacillus reuteri* 6475	Oh et al., 2019
Fructose	Unspecified	*Lactobacillus reuteri* 55730	Oh et al., 2019
Nestea	933W Stx	*Escherichia coli* MG1655	Pierzynowska et al., 2018
β-Lactam antibiotics	80α and φ11	*Staphylococcus aureus* RN27 and RN451	Maiques et al., 2006
Quinolone antibiotics	Stx2	*Escherichia coli* O157:H7	Zhang et al., 2000
Ciprofloxacin	Unspecified	*Enterococcus faecalis* V583ΔABC	Rossmann et al., 2015
Short-chain fatty acids	LRΦ1 and LRΦ2	*Lactobacillus reuteri* 6475	Oh et al., 2019
Short-chain fatty acids	Unspecified	*Lactobacillus reuteri* ATCC 55730	Oh et al., 2019
Short-chain fatty acids	ΦTP901	*Lactococcus lactis* NZ9000	Oh et al., 2019
Gastrointestinal transit	LRΦ1	*Lactobacillus reuteri* 6475	Oh et al., 2019
Quorum sensing	Unspecified	*Enterococcus faecalis* V583ΔABC	Rossmann et al., 2015
High temperature	λts type II	*Escherichia coli*	Horiuchi and Inokuchi, 1967
Hydrogen peroxide	Lambdoid	*Escherichia coli* MG1655	Łoś et al., 2009
Inflammation	SopEΦ	*Salmonella enterica* Typhimurium SL1344	Diard et al., 2017
Nitric oxide	Stx2	*Escherichia coli* O157:H7	Ichimura et al., 2017
Acyl-homoserine lactones	Lambda	*Escherichia coli* BW25113	Ghosh et al., 2009

### The Mechanisms of Prophage Activation

The induction of prophages by various factors are mostly related to the RecA protein and SOS response of bacteria (Diard et al., 2017; Oh et al., 2019). The commensal relationship between prophages and hosts is mainly supported by the silence of the SOS system, which is a pathway mainly responsible for bacterial DNA damage response (Au et al., 2005; Kreuzer, 2013). The SOS system in bacteria coordinates cellular response to DNA damage via linkage action between RecA protein and LexA repressor (Friedberg et al., 2005). Naturally, the expression level of SOS regulon genes are limited by inactive promoter regions that are occupied by LexA. Upon DNA damaged, RecA protein forms active RecA filament termed activated RecA, on single-stranded DNA, and acts as a coprotease which catalyzes the self-cleavage reaction of LexA in DNA-free form, probably through reducing the pK_*a*_ of a key lysine residue (Little, 1991; Luo et al., 2001; Matej et al., 2011). Consequently, SOS genes expression are up-regulated. By using single-molecule imaging techniques for live cells, it was proved that the RecA protein was sequestered in storage structures until DNA damage happened, and early SOS-signaling complexes were formed subsequently (Ghodke et al., 2019). The polymerases ImuB and ImuC acting as factors involved in the regulation of SOS system were co-transcribed under the control of LexA in *Pseudomonas aeruginosa* (Luján et al., 2019). The *exo-xis* region of lambdoid bacteriophages and OxyR regulator influenced prophage maintenance and induction (Bloch et al., 2013; Licznerska et al., 2015). Importantly, production of viral progeny was dependent on inactivation of phage repressor, expression of phage antirepressor, or activation of specific mutagenesis proteins (for example, Umud protein) (Carrasco et al., 2016; Diard et al., 2017; Ichimura et al., 2017; Silpe and Bassler, 2019). Various phages replicated either by transposition or episomally once induced, and virion particles were then assembled and packaged with phage DNA by endonucleolytic enzymes (reviewed in Howard-Varona et al., 2017).

Spontaneous DNA damage and low levels of SOS genes expression were observed in wild-type bacterial cells, which might be associated with mismatches during DNA replication and the DNA “damage-up” proteins (DDPs) (Mccool et al., 2004; Xia et al., 2019). Moreover, low-fidelity polymerase pol VICE391 (RumA’2B) encoded by conjugative transposone R391 could further promote higher levels of spontaneous SOS mutagenesis, partly because of the longer binding time of RumB to genomic DNA (Walsh et al., 2019). While the mechanisms of some prophage inducers (for example, diet, clove, stevia, grapefruit seed extract, and aspartame)-mediated prophage activation have remained poorly understood, inflammation (e.g., reactive nitrogen species, reactive oxygen species, and hypochlorite), dietary fructose, nitric oxide, SCFAs, β-lactam antibiotics, and hydrogen peroxide have been shown to induce phage production by activating the SOS response in *Salmonella enterica* Typhimurium (*S*.Tm) SL1344, *L. reuteri* 6475, EHEC O157, *Lactococcus lactis* NZ9000, *Staphylococcus aureus*, and *Escherichia coli* MG1655, respectively (Maiques et al., 2006; Howe et al., 2016; Diard et al., 2017; Ichimura et al., 2017; Bloch et al., 2018; Kim and Bae, 2018; Oh et al., 2019; Boling et al., 2020).

In general, high-level expression of SOS genes has been considered as a consequence of the decrease in LexA repressor levels, whereas the decreasing signal from RecA protein or LexA allows the SOS system to be shut off. The activation of SOS system induces production of active phages.

However, it is worth noting that in addition to the SOS response, some alternative pathways can induce the activation of prophages. In some lysogenic cells (for example, *Staphylococcus aureus* and *Escherichia coli*), spontaneous prophage induction does not require SOS response, but depend on phage repressor cleavage or cell density (Ghosh et al., 2009; Haaber et al., 2016). Prophages encoding Shiga toxin 2 were still inducible in Δ*recA* mutants, indicating that there may be multiple causes for prophage induction (Colon et al., 2016). The SOS-independent prophage induction was well demonstrated in a co-culture system, in which acyl-homoserine lactones (AHL) produced from *Pseudomonas aeruginosa* triggered lambda phage production in *recA*-deficient *Escherichia coli* (Ghosh et al., 2009). The specific counteraction of xenogeneic silencers (XS) including H-NS etc. can also modulate prophage activity. For example, double depletion of the MvaT and MvaU proteins, belonging to the H-NS family, activated prophage Pf4 in *Pseudomonas aeruginosa* PAO1 (Li et al., 2009). Moreover, inhibition of the transcription termination factor Rho led to induction of lytic cycle of prophages in *Escherichia coli* (Menouni et al., 2013). These examples highlight the diversity mechanisms of prophage activation.

### The Spreading of Induced Active Phages in the Intestine

**FIGURE 1:**
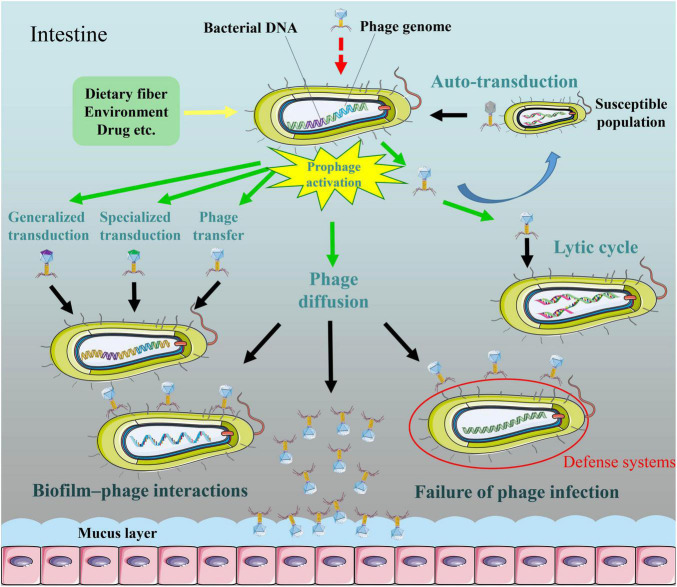
The diffusion of induced active bacteriophages in the intestine. Activators may activate SOS system in lysogenic bacteria and then induce production of active phages. Phages released from a subpopulation of lysogenic bacteria are able to capture DNA from competitor cells, and subsequently transfer to remaining population, the process which termed “auto-transduction.” Some temperate phages exhibit lysogenic conversion or transduction (generalized transduction or specialized transduction) in specific environments. Temperate phages can infect specific bacteria for lytic cycles. Phages are adapted for attaching to bacteria and forming multicellular communities in a biofilm environment. Bacterial defense systems are able to restrict temperate phage acquisition and lytic phage predation. Phages have more potential to enrich in mucus layer relative to the adjacent environment.

Temperate phages exhibit lysogenic conversion or transduction in specific environments (Figure 1). Transduction typically occurs at low frequency and comes in two varieties: specialized transduction (flanking bacterial DNA of prophages is excised and packaged into the capsid) and generalized transduction (random bacterial or plasmid DNA fragments are accidently packaged into the capsid) (Touchon et al., 2017). Whereas all phages have the potential for transduction, transduction rates vary significantly between phages (Kenzaka et al., 2007). Phages utilizing the headful packing strategy showed relatively high transduction rates (Wu et al., 2002). In addition, the ratio of phage particles to potential bacterial hosts (phage: bacteria ratio/multiplicity of infection) may change transduction frequency. For example, as the phage: bacteria ratio increased from 0.01 to 1, the frequency of plasmid pNZ8048 transduction by bacteriophage 5171F increased first and then decreased (Marcelli et al., 2020). It is commonly assumed that most transducing phages are incomplete/defective and thus transduction can be regarded as one-shot event (Touchon et al., 2017). Lysogenic conversion can be associated with specialized transduction in certain cases if the prophage excision from a donor cell is not precise. Inflammatory factors reactive oxygen species and reactive nitrogen species etc. could induce the SOS response of *S*.Tm and enhance the expression of phage (SopEΦ) anti-repressor Tum, and as a result, free phages were produced (Diard et al., 2017). SopEΦ, the temperate phage belonging to P2 family, was observed with a low degree of lysogenic conversion from *S*.Tm SL1344 to *S*.Tm ATCC14028S in the gut lumen of mice without inflammation (Diard et al., 2017). However, intestinal inflammation and disease triggered 10^5^-fold SopEΦ transfer within 3 days in that study. During an epidemic, the bacteriophage *sopE* gene spread extensively in monophasic *S*.Tm (Petrovska et al., 2016). Therefore, prophage activation drives lysogenic conversion. Furthermore, β-lactam antibiotics facilitated horizontal transfer of phages in *Staphylococcus aureus* (Maiques et al., 2006). As a matter of fact, use of antibiotics is tightly linked to efficient phage transfer in a few pathogenic bacteria (for example, Shiga toxin-producing *Escherichia coli*, *Clostridium botulinum*, and *Salmonella*) (reviewed in Tamang et al., 2017).

A study indicates that phages released from a subpopulation of lysogenic *Staphylococcus aureus* were able to capture DNA from competitor cells, and subsequently transferred to remaining population, the process which termed “auto-transduction” (Figure 1; Haaber et al., 2016). Nevertheless, it remains unclear whether auto-transduction is restricted to *Staphylococcus aureus.* Both transduction and auto-transduction facilitate the transfer of bacterial DNA to sensitive population.

Certain bacteriophages possess a dual strategy for perpetuation (Wadhwa, 2017; Laganenka et al., 2019). Induced active phages can reproduce in a short-time lytic life (Figure 1). It should be also mentioned that the filamentous single-stranded deoxyribonucleic acid (ssDNA) phages of the subclass *Inoviridae* uniquely follow a productive chronic life cycle in which the maturing phage progeny is secreted through the cell envelop without killing the host (reviewed in Sausset et al., 2020). The small-molecule communication system termed “arbitrium” system, quorum sensing, metabolic state of bacteria, and multiplicity of infection etc. controlled lysis–lysogeny decision of bacteriophages (Erez et al., 2017; Wadhwa, 2017; Laganenka et al., 2019). Depending on specific phage receptors present on the cell surface of target bacteria, some of the phages were only capable of infecting a single strain, while others could infect bacteria belonging to different genera. Phage receptors on the bacterial surface include lipopolysaccharide, pili, flagella, membrane proteins (for example, porins) and so on (reviewed in Dy et al., 2014).

Since one bacterium is capable of releasing hundreds of phage particles, prophage induction may increase the struggle between bacteriophage and bacteria, and drive bacterial anti-phage system evolution. Indeed, at each stage of phage infection process, bacteria have evolved anti-phage approaches, such as adsorption and DNA injection inhibition, abortive infection, toxin-antitoxin, and CRISPR-Cas systems (reviewed in Dy et al., 2014). In recent years, some novel anti-phage defenses were reported, such as defense island system associated with restriction-modification, the chemical anti-phage defense system, and prophage defense strategy (Figure 1; Kronheim et al., 2018; Ofir et al., 2018; Ragunathan and Vanderpool, 2019). Evolutional arm race also promotes phages to develop evasion mechanisms to escape bacterial resistance strategies (reviewed in Dy et al., 2014). Overall, prophage induction in the intestine may greatly enhance phage-bacterium interactions.

Despite it is known that intestine contains vast numbers of free phages and microorganisms, the mechanisms of long-term coexistence of phages and bacterial population remain largely unexplored. The study of Chaudhry et al. (2019) illustrated that mucoidy was related to this biological phenomenon, thereby ensuring a stable bacterial density in intestine. A recent study demonstrated that phage-inaccessible sites in the mucosal layer protected bacteria against phage predation, suggesting that coexistence of phages with phage-susceptible bacteria benefits from the heterogeneous biogeography of microbe (Lourenço et al., 2020). Active phages were adapted for attaching to bacteria and forming multicellular communities in a biofilm environment (Simmons et al., 2018). Biofilm–phage interaction life is likely to be ubiquitous as a natural feature of bacteriophages (Figure 1). It should be noted that phages have more potential to enrich in mucus layer relative to the adjacent environment (Figure 1; Barr et al., 2013). This bacteriophage adherence to mucus model occurs via binding interactions between phage Ig-like protein domains (for example, Hoc) and glycan residues of mucin glycoproteins. In addition, the intrusion of intestinal phages to other body tissues (for example, the kidney and liver) by bloodstream was not only caused by impaired integrity of the intestinal epithelium due to intestinal inflammation, but also occurred in some normal physiological conditions (reviewed in Chatterjee and Duerkop, 2018). Beyond these examples, induced active phages may have more extensive diffusion pathways.

## Effects of Prophage Activation on Physiological Characteristics of Bacteria and Intestinal Health

### Effects of Prophage Activation on Physiological Characteristics of Bacteria

In recent years, prophage induction (or excision) in the intestine have attracted great attention for its potentially important functions. In a phenomenon called reversible active lysogeny, the excision of prophage of the *Listeria monocytogenes* genome during infection would reactivate the host *comK* gene for immune evasion, avoiding phagosomes in macrophage cells, but this process didn’t produce progeny virions (Rabinovich et al., 2012). Excision of cryptic prophages that affected the expression of transfer-messenger RNA and biofilm formation improved *Shewanella oneidensis*’s adaptability to low temperature (Zeng et al., 2016). The results of Nedialkova et al. (2016)’s study indicated that activation of lambdoid prophages in *S*.Tm was necessary to release the bacteriocin Colicin Ib that conferred a competitive advantage for bacterial hosts in the competition against Colicin Ib-susceptible competitors. Previous reports showed that spontaneously induced prophages could affect the living strategy of bacteria, deliver fitness benefits to the host, and increase virulence of specific pathogens (Nanda et al., 2015; Chakraborty et al., 2018; Balasubramanian et al., 2019). Given the ability of bacteriophages to switch their infection modes, prophage activation is a strategy to kill competitors once the prophages are induced and released, assuming that not all the lysogenic cells will die (Haaber et al., 2016). With the above mentioned results, it can be seen that prophage activation (or excision) is crucial for bacterial survival and adaptability in response to complex and unfavorable living environment.

Lysogenic conversion and horizontal gene transfer by transduction mediated by prophage activation promote bacterial virulence and pathogen evolution as well as antibiotic resistance encoded by accessory loci, which allow bacteria to rapidly adapt to new hosts. Livestock-associated *Staphylococcus aureus* CC398 strains gained the ability to produce staphylokinase, enterotoxin A, and Panton-Valentine leucocidin (PVL) toxin by lysogeny with immune evasion cluster (IEC)/PVL-converting bacteriophages, utilizing the temperate phages P240, P282, P630, and P1105 (Kraushaar et al., 2017). *Streptococcus thermophilus* phage DLP4 had lysogenic transformation ability which could help host strains acquire antibiotic resistance (Peters et al., 2019). The transfer of prophage Φ3538 Δstx2:cat contributed to converting atypical enteropathogenic *Escherichia coli* (aEPEC) to EHEC (Eichhorn et al., 2018). A study showed that lysogenic conversion by filamentous prophage CTXΦ in *Vibrio cholerae* caused the spread of virulence genes (i.e., cholera toxin gene *ctxAB*) in non-pathogenic bacteria, which made these strains become toxic (Waldor and Mekalanos, 1996). Prophages can transfer not only their own genomes, but also bacterial DNA. Transducing phages may lost part or all of own genome and phage capsid allows to package appropriate length of hosts DNA that is even longer than phage genome (reviewed in Touchon et al., 2017). Prophage-mediated lateral transduction was capable of transferring large metameric spans of the *Staphylococcus aureus* genome (Chen et al., 2018). The spread of virulence genes can be strongly facilitated by Viunalikevirus-relevant generalized transduction (Matilla et al., 2014). Transduction bias in transferred DNA may be present. This is because some mobile genetic elements such as phage-inducible chromosomal islands (PICI) tend to hijack transduction for their priority transmission (Fillol-Salom et al., 2019). The PICI-encoded RppC (for redirecting phage packaging) protein bound to the phage terminase TerS to form a heterocomplex that only recognized PICI genome while excluding phage DNA, thus packaging PICI into a newly formed phage head and furthering the dissemination of pathogenic features among Gram-negative bacteria. In particular, auto-transduction of *Staphylococcus aureus* helped to obtain DNA from competitors through phage transducing particles, which conferred potentially beneficial traits (i.e., antibiotic resistance) to remaining, prophage-containing population (Haaber et al., 2016). Overall, a set of data suggests that temperate bacteriophages play important roles in bacterial adaption and evolution (via lysogenic conversion, transduction, or auto-transduction).

However, prophage induction exerts negative effects on their bacterial host in most cases. Prophage induction triggered by stochastic fluctuations or environmental stressors tend to resume the lytic cycle and subsequent lysis of the lysogen. During the gastrointestinal transit, phage production was found to negatively impact on the survival of *L. reuteri* (Alexander et al., 2019; Oh et al., 2019). Furthermore, in IBD patients, prophage activation had been associated with the depletion of *Faecalibacterium prausnitzii* (*F. prausnitzii*), a main commensal bacteria in the human gut (Cornuault et al., 2018). Collectively, the survival of intestinal bacteria is challenged by unstable prophages that can kill the hosts at any time.

On balance, host’s fitness, metabolic repertoire, and ecological evolution can be changed with prophage induction.

### Effects of Prophage Activation on Intestinal Health

The effects of prophage activation on intestinal health are reflected in two aspects. In terms of direct microbial-related effects, prophage activation in an inflammatory environment could aggravate *Salmonella*-induced diarrhea and intestinal microbiota dysbiosis (Diard et al., 2017; Cornuault et al., 2018). In mouse infected by EHEC, prophage induction that didn’t produce active virus particles was essential for the production of Shiga toxin and lethal disease (Balasubramanian et al., 2019). Several studies have investigated the relationship between gut viriome composition and pathological state of individuals. During inflammatory disease, increased intestinal phage population was found, especially the phages specific to pathobiotic hosts, suggesting enhanced mortality of lysogens (Duerkop et al., 2018). A study reported more abundant *F. prausnitzii* phages in IBD than in those of healthy mice (Cornuault et al., 2018). Notably, it is well known that the lack of gut microbiota homeostasis is highly correlated with several diseases (for example, Crohn’s disease and ulcerative colitis). Taken together, one may speculate that the prevalence of prophage activation during IBD results in lysis of the normal intestinal flora or bacterial symbionts, thereby aggravating dysbiosis. Prophages are potentially as natural modulators of the bacterial dynamics in the human intestine and thus in preventing/establishing gut microbiota dysbiosis. Moreover, liberating cellular contents can be supplied as nutrients to neighboring bacteria (Nanda et al., 2015). On the other hand, due to the activation of prophages, a large number of progeny phage particles are produced in the intestine. Gut bacteriophages were able to pass through the epithelial cell layer of the intestine. This fact was evidenced by either *in vitro* transwell system or *in vivo* orally administered experiments (Reynauda et al., 1992; Nguyen et al., 2017). Since bacteriophages share some common features with viruses that target mammalian cells, they can be recognized by innate host receptors (for example, members of the Toll-like receptor family). There is ample evidence about direct interactions between intestinal phages and host immune cells (dendritic cells, B cells, and monocytes) recently reviewed by Seo and Kweon (2019) and Federici et al. (2020), which are shown to increase the production of antiviral cytokines (for example, chemokines, interferon-γ, and interleukin-12) and potential contamination of endotoxin (for example, lipopolysaccharide). Consistently, according to results of Gogokhia et al. (2019), expansion of bacteriophages might induce expression of cytokines, aggravate intestinal inflammation, heighten immune response, and exacerbate murine colitis. The important point is that not all phages stimulate such immune response (for example, T4 phage) (Miernikiewicz et al., 2013). Indeed, phages from the ulcerative colitis patients induced secretion of higher amount of interferon-γ than those from health controls, suggesting the critical role of specific bacteriophages in immunomodulation (Gogokhia et al., 2019). By contrast, Vitetta et al. (2018) noted that bacteriophages displayed effects in protecting against commensal pathobionts, maintaining intestinal homeostasis, and controlling bacterial concentrations in gut. Mucus-adherent phages could form an antibacterial barrier that actively protect the mucosal surfaces of the intestinal epithelium from bacterial infection (Barr et al., 2013). Released temperate phages may modify gut bacterial communities by multiple life cycles including lysing competitive or sensitive cells, lysogenizing other bacteria as well as continuous and complex phage-bacterium interactions (reviewed in Sausset et al., 2020). Prophage activation induced by antibiotics (for example, ciprofloxacin) promoted transfer of Stx2 prophage and thus non-pathogenic bacteria may produce toxin in the gut and stimulate the host immune response, an example of lysogenic conversion demonstrating the indirect modulation of host immunity by phages (Zhang et al., 2000). This observation suggests that phage transfer could have an ultimately downstream influence on bacterial-mammalian interactions. Beyond that, cascading effects were observed in non-susceptible bacteria via interbacterial interactions when specific phage predation occurred (Hsu et al., 2019). Hence one can see that prophage activation may have a systemic effects. Currently, the effects of bacteriophages on gut health did not show a consistent pattern in previous studies and phages may play dual roles (positive and negative aspects) in gut health. Impact of prophage activation may dependent on the activation response amplitude, prophage types, specific intestinal environments (healthy intestinal inter-environment and IBD, etc.), and bacterial community composition.

### Phage Therapy by Regulating Prophage Activation

Lytic phages have become the unanimous first choice for phage therapy, avoiding increased bacterial virulence mediated by lysogenic conversion. Even so, people have never stopped looking for novel approaches to phage therapy, such as the use of temperate phages against bacterial infection (Monteiro et al., 2019). Since prophage activation and subsequently potential phage transfer are common and important physiological phenomenon in bacteria (as mentioned earlier), we can expand our arsenal against the persistent threat of drug resistance of bacteria by utilizing advanced biotechnologies and regulating prophage activation properly. Below we discuss phage therapy strategies that rely on the process of prophage activation.

**FIGURE 2:**
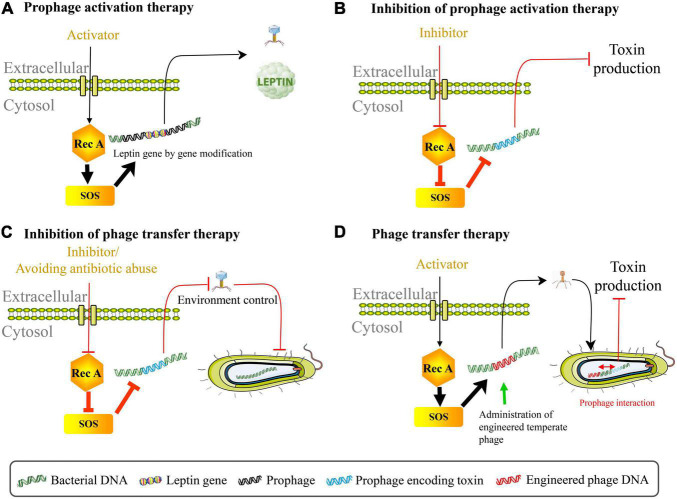
Phage therapy by regulating prophage activation. **(A)** Prophage activation therapy. The leptin protein can be released with prophage activation using gene editing technology to insert the leptin gene into native phage genome in *Lactobacillus reuteri* VPL1014. **(B)** Inhibition of prophage activation therapy. Suppressing the spontaneous induction of virulence-carrying prophage in *Escherichia coli* O157:H7 can reduce the production of toxin. **(C)** Inhibition of phage transfer therapy. Preventing lysogenic conversion among pathogens includes following aspects: block bacterial SOS response pathway through using RecA protein inhibitor, avoid antibiotic abuse, interrupt SOS-to-prophage activation signal, and regulate intestinal environment etc. **(D)** Phage transfer therapy. The administration of temperate engineered phages inhibits toxin production caused by virulence-carrying prophages in EHEC through phage transfer and prophage interaction.

The property of induction of prophage genomes provides new avenues for clinical treatment. By using gene editing technology to insert the leptin gene into native phage genome of *L. reuteri* VPL1014, the leptin protein was released with prophage activation, implying that prophages can serve as a microbial therapeutic delivery target (Figure 2A; Alexander et al., 2019). The application of dietary prophage inducers opens a new path for altering the gut microbiome (Boling et al., 2020). It is also possible to genetically modify the phage genome involved in prophage maintenance, and then start lytic cycles in pathogens (Zhang et al., 2013). In contrast, cinnamon oil eliminated RecA protein, polynucleotide phosphorylase, and poly(A) polymerase in *Escherichia coli* O157:H7, thereby inhibiting prophage activation and Shiga toxin production (Figure 2B; Sheng et al., 2016). Under oxidative stress conditions, suppressing induction of the prophage in Shiga toxin-producing *Escherichia coli* using some derivatives (for example, CM092, CM032D, and CM3186B) of quinolone, indazole, indenoindole, triazole, carbazole, and ninhydrine reduced bacterial virulence (Bloch et al., 2018). This inhibitory effect on prophage induction was achieved by increasing the expression of genes responsible for encoding *c*I repressor and reducing the expression of oxidative stress genes as well as phage lysis genes. One mechanistically particular example is suppressing Shiga toxin production in EHEC via activation of guanosine tetraphosphate (ppGpp) synthesis (Nowicki et al., 2014). These facts provide the guidance for the proper use of prophage activation regulation therapy during treatment for bacterial intestinal disease.

Increased bacterial virulence and antibiotic resistance that may be caused by phage lysogenic conversion pose great threats to the human’s health, and therefore, prevention of bacterial lysogenic conversion is necessary for disease prevention and medical treatment. It can be approached from the following five aspects: (1) Block bacterial SOS response pathway; (2) Interrupt SOS-to-prophage activation signal; (3) Reduce bacterial inflammatory properties or relieve intestinal inflammation with external intervention; (4) Vaccination against gut disease; (5) Avoid the abuse of antibiotics (Figure 2C). In recent years, the short first in class α-helical peptide, phenolic compound N-acetylcysteine, 5-amino-1-(carbamoylmethyl)-1H-1, 2, 3-triazole-4-carboxamide scaffold, zinc, phthalocyanine tetrasulfonic acid, fermentates from probiotic strains, and p-Coumaric acid were found to inhibit RecA protein activities, thereby blocking the SOS system (Alam et al., 2016; Bunnell et al., 2017; Yakimov et al., 2017; Selwood et al., 2018; Ojha and Patil, 2019; Prazdnova et al., 2019; Rodríguez-Rosado et al., 2019). RecA protein inhibitor bind to the L2 loop through the ssDNA site on the epithelium (Bellio et al., 2017). Diard et al. (2017) found that during inflammation, phage transfer was blocked when the *tum* gene encoding phage antirepressor was deleted (signal interruption from bacterial SOS response to prophage induction). Their results also showed that avirulent *S*.Tm variants constructed by destroying type III secretion system 1 and 2 couldn’t trigger inflammation and therefore reduced rates of phage transfer. More studies are expected to explore whether functional compounds such as *Pteris multifida*, *Cortex phellodendri*, and astragalus polysaccharide which could attenuate *Salmonella*-induced intestinal inflammation can inhibit phage-mediated horizontal gene transfer (Yin et al., 2018; Dong et al., 2019). Importantly, mucosal vaccination prevented inflammation disease and limited inflammation-dependent lysogenic conversion by phages, indicating that vaccination may be one of an effective strategy for blocking pathogen evolution (Diard et al., 2017). Since antibiotics may not only exacerbate virulence via inducing toxin-encoded prophages but also promote horizontal dissemination of virulence factors, it is critical to control the abuse of antibiotics and explore novel therapeutic strategies in the treatment of bacterial infection (for example, phage therapy) (Zhang et al., 2000; Maiques et al., 2006; Monteiro et al., 2019). Intriguingly, bacterial dormancy might curb phage epidemics (Jackson and Fineran, 2019). These approaches may also provide reference to the inhibition of phage transduction/auto-transduction that potentially accelerate pathogen evolution. Contrarily, it has to be mentioned that recent discovery of bacteriophage transfer therapy that used engineered λ phages overcame resistance and reduced the production of Shiga toxin encoded by the virulence-expressing prophages in EHEC through transcriptional repression strategy (Figure 2D; Hsu et al., 2020). In this case, lysogenic conversion of engineered λ phages in bacterial communities rather than anti-bacterial action enhanced the curative effect of stable neutralization of virulent *Escherichia coli*. Therefore, phage transfer may act as a double-edged sword for gut health.

Despite prophage as a novel potential therapeutic agent is important and promising, it still remains largely unexplored. Scientific evidence for prophage activation regulation therapy is relatively scarce. There are several major concerns about the application of prophage genome as targets for phage therapy. First, compared with phage therapy using strictly lytic phages, prophage activation regulation therapy is limited to functional prophage genome (i.e., prophage encoding specific function or easily genetically modified and activated). Second, considering complex internal environment of the intestine, therapeutic effects of prophage activation regulation therapy may be affected by unknown factors. Third, much less is known about specialized transduction, generalized transduction, and auto-transduction that may transfer resistance and virulence determinants to intestinal beneficial bacteria. Finally, due to lack of understanding of the most phage genes, unexpected and undesirable events may happen after gene modification. A better understanding of basic gene function of phages is urgently needed.

### Research Prospects

Over the last years, a large number of studies have shown the effects of multiple factors (e.g., exercise, physiological changes, diets) on the alteration of gut microbiome (Muegge et al., 2011; Jensen et al., 2020; Mahizir et al., 2020). However, the research on enteroviruses is still relatively backward. Although the effect of certain drugs or bacterial activities on prophages have not been comprehensively clarified, it has been proven that they can trigger SOS response in bacteria. For example, continued exposure to sublethal doses of ciprofloxacin increased competitive fitness of *Pseudomonas aeruginosa* through SOS pathway (Torres-Barceló et al., 2015). The uptake of DNA from prey cells by *Acinetobacter baylyi* using type VI secretion system resulted in the upregulation of the SOS response and extensive filamentation (Lin et al., 2019). Antimicrobial peptide (AMP) including periplanetasin-2 and bac8c induced *Escherichia coli* apoptosis-like death via reactive oxygen species (ROS) relating to the participation of RecA protein and the SOS system (Lee and Lee, 2019; Lee et al., 2019). Bacterial ROS production and SOS response could increase bacterial mutagenesis and resistance (for example, fluoroquinolones) (Rodríguez-Rosado et al., 2019). The deleterious effects such as mutagenesis and cell death in *Bacillus subtilis* caused by hexavalent chromium were counteracted by SOS response system in a RecA protein-dependent manner (Santos-Escobar et al., 2019). DNA gyrase depletion in *Mycobacterium tuberculosis* could activate RecA/LexA-mediated SOS response by inducing persistent subpopulations (Choudhary et al., 2019). Based on the phage production mechanisms discussed above, it can be speculated that these drugs or bacterial activities may induce more active phages in the intestine due to the activation of bacterial SOS system. The work of Oh et al. (2019) confirmed that SCFAs served as an activator of RecA protein in *L. reuteri*. Small molecules together with other metabolites produced in bacteria in relationship to the activation of prophages deserve in-depth and extensive research. It is a remarkable fact that some strains, such as *Acinetobacter baumannii* and *Acinetobacter baylyi*, do not have a homolog of LexA, suggesting that we should pay particular attention to prophage activation of these strains (Hare et al., 2014; Nguyen et al., 2019). The distribution of released phages is specific in the medium containing different inducers or cultivating in different stimulative environment, which implies the particularity of phage production dynamics (Fang et al., 2017; Oh et al., 2019). The types of temperate phages, prophage gene length, the interaction with bacterial genome, the interaction with other prophages, the SOS response intensity, special regulatory factors, integration site, and physiological characteristics of bacterial phages may play important roles in regulating of the prophage response during bacterial global stress (Tang et al., 2017). Currently, the knowledge of coexistence of free phages and bacterial community in gut is limited. It needs to be further studied whether or not there is an ecological niche competition between active phages and bacteria. Few studies comprehensively and systematically investigated how prophage activation induced by exogenous interference affect intestinal health. The roles of gut temperate phages and the potential biological significance of the increased number of bacteriophages in the intestines of patients such as IBD and CDI remain largely to be explored. Classification and susceptible hosts of intestinal temperate phages are weakly unknown, which are crucial problems for the further understanding of intestinal phages. Although understanding prophage activation of pathogenic bacteria may be of greater significance, we have little knowledge about the activation and transfer of prophages in non-pathogenic gut bacteria.

With the in-depth understanding of phages and the development of biotechnology, temperate phages are introduced into phage therapy. Prophage activation regulation therapy has broad application prospects in terms of the bacterial disease treatment. It inspires us that dietary intervention, medicine use, genome editing, genetically engineered bacteriophages, and environmental control which can potentially regulate the dynamic changes of prophages can be used to maintain the intestinal health. In addition, cryptic (defective) prophage excision that cannot lyse their hosts and produce active phages is a potentially promising target for the phage therapy development (Wang and Wood, 2016). Many studies have investigated roles of prophages in their bacterial hosts, wherein, some (cryptic) prophages were considered as being mutualistic (Obeng et al., 2016). A wealth of evidence has revealed that cryptic prophages make contributions in some beneficial phenotypes (for example, virulence, antibiotic resistance, and antibiotic tolerance) in bacteria and has been thoroughly reviewed by Wang and Wood (2016). Therefore, once the beneficial cryptic prophages are excised by using inducers or genetically modified methods, the adaptive advantage of host obtained from prophages is likely to be lost. Cryptic prophage excision may also reduce the cell viability via enhancing the expression of cell lysis genes (for example, *alpA and intD*) (Wang et al., 2009). However, it should be noted that cryptic prophage excision has a low probability of occurrence under various environments, which may hinder its application in bacterial disease (Sozhamannan et al., 2006). In short, we are looking forward to making a breakthrough in prophage activation/excision regulation therapy in the future.

## Conclusion

Intestinal prophages can be activated by a variety of factors, including diet, antibiotics, certain bacterial metabolites, gastrointestinal transit, inflammatory environment, intestinal temperature change, oxidative stress, and quorum sensing, etc. Released active phages may experience several different life status, including lysogenic conversion, transduction, auto-transduction, lytic cycles, mucoidy, biofilm–phage interaction, and bacteriophage adherent to mucus model etc. Effects of prophage induction on bacterial host and gut health have both positive and negative sides. Thus, as part of the multidimensional strategies for bacterial disease, prophage activation regulation therapy is flexible. Nevertheless, efforts should been made to know more mechanisms about how prophage induction happened, dynamic transformation of prophages, and the role of prophage activation in gut health. While lytic phages are likely to remain the main choice for phage therapy, considering the importance of prophages in bacterial function and evolution, prophage activation regulation therapy is worthy of thorough study by researchers.

## Author Contributions

JH: writing–original draft preparation, visualization, and conceptualization. HY: writing–review and editing. SW: conceptualization. JW: conceptualization, funding acquisition, and project administration. DH: supervision, project administration, writing–review and editing, and funding acquisition. All authors have approved this work for publication.

## Conflict of Interest

The authors declare that the research was conducted in the absence of any commercial or financial relationships that could be construed as a potential conflict of interest.

## Publisher’s Note

All claims expressed in this article are solely those of the authors and do not necessarily represent those of their affiliated organizations, or those of the publisher, the editors and the reviewers. Any product that may be evaluated in this article, or claim that may be made by its manufacturer, is not guaranteed or endorsed by the publisher.
